# Tracking the dynamic breakdown of contextual coherence in schizophrenia using language models

**DOI:** 10.3389/fpsyt.2026.1848956

**Published:** 2026-06-01

**Authors:** Seunghyong Ryu, Ju-Wan Kim, Min Jhon, Young-Chul Chung, Seok Jun Kim, Suehyun Lee, Jae-Min Kim, Sung-Wan Kim

**Affiliations:** 1 Department of Psychiatry, Chonnam National University Medical School, Gwangju, Republic of Korea; 2 Department of Psychiatry, Jeonbuk National University Medical School, Jeonju, Republic of Korea; 3 Department of IT Convergence, Graduate School, Gachon University, Seongnam, Republic of Korea; 4 Department of Computer Engineering, College of IT Convergence, Gachon University, Seongnam, Republic of Korea; 5 Mindlink, Gwangju Bukgu Community Mental Health Center, Gwangju, Republic of Korea

**Keywords:** disorganized speech, natural language processing, schizophrenia, surprisal, temporal dynamics

## Abstract

Disorganized speech is a core clinical feature of schizophrenia, reflecting disruptions in contextual integration. To objectively quantify the unfolding nature of natural speech, this study investigated the dynamic temporal trajectory of contextual coherence breakdown using sequential surprisal derived from autoregressive language models. We analyzed transcripts from 249 patients with schizophrenia and 159 matched healthy controls across eight diverse speech tasks. Using two Korean small language models (Polyglot-ko-5.8b and Kanana-1.5-8b-base), we calculated token-by-token surprisal, a measure of lexical predictability, from 100-token utterances. To account for the inherent volatility of autoregressive models at speech onset, temporal divergence of surprisal trajectories between groups was analyzed from tokens 11 to 100 using generalized additive mixed models. Patients exhibited progressively divergent surprisal trajectories as discourse unfolded, reflecting a breakdown in contextual predictability. Emerging early in the discourse, these deviations consistently intensified by tokens 50 to 70, indicating a rapid deterioration in the capacity to sustain global contextual constraints even within a short utterance. Both models also detected significantly higher overall mean surprisal in patients. Furthermore, this temporal divergence was most pronounced during unstructured narrative tasks, which lack the external visual cues of projective tasks. These findings provide quantitative evidence that language anomalies in schizophrenia are dynamic phenomena rapidly unfolding within a short discourse. By capturing precise temporal intervals of contextual disruption and paralleling clinical descriptions of disorganized discourse, this research highlights the utility of temporal natural language processing metrics in elucidating the psychopathology of schizophrenia.

## Introduction

Linguistic impairments, frequently manifesting as formal thought disorder, are core features of schizophrenia (1). Recent evidence indicates that these deficits pervasively affect multiple interacting language domains, including semantics, prosody, phonology, and morphosyntax (2). This breakdown clinically manifests as disorganized speech, tangentiality, and impaired coherence, reflecting cognitive failures in semantic integration and executive functioning (3). Despite their diagnostic prominence, objectively quantifying these disruptions remains challenging, as conventional subjective rating scales often lack sensitivity to dynamic and subtle linguistic deviations (4).

To address these methodological limitations, recent psychiatric research has increasingly utilized Natural Language Processing (NLP) (5). Language models encode the statistical regularities of normative language, providing a standardized computational baseline for evaluating clinical speech (6). These models successfully operationalize clinical abnormalities as measurable probabilistic divergences, frequently utilizing metrics of overall semantic and syntactic predictability (7). Specifically, Generative Pre-trained Transformer (GPT) architectures process language autoregressively, calculating the probability of the next token sequentially, conditioned on preceding context (8).

This unidirectional, autoregressive mechanism shares conceptual parallels with human language processing (9, 10). At utterance onset, the lack of established context inherently demands greater cognitive loading to predict the contextual trajectory (11). However, as words accumulate, context solidifies, rendering subsequent language more predictable and reducing cognitive demand (12). GPT models can capture this dynamic predictability via token-level “surprisal, ” defined as the negative log probability of a token given prior context (13). High surprisal indicates an unexpected word, whereas low surprisal reflects strong contextual alignment (14).

Applying this sequential probabilistic framework provides a novel approach for investigating the temporal dynamics of discourse breakdown in schizophrenia (5). Clinically, patients may initially generate coherent short phrases with normative predictability and contextual continuity. However, as an utterance progresses and demands sustained integration of an expanding context, patients frequently deviate from the topic, resulting in contextual disruption (15). This progressive contextual disruption suggests that linguistic anomalies are not uniformly distributed across a speech sample. Instead, they emerge dynamically as discourse lengthens and the cognitive demands of maintaining global context exceed the patient’s capacity (16).

Therefore, this study investigated the dynamic trajectory of disorganized speech in a Korean-speaking cohort with schizophrenia using GPT-derived token-level surprisal. By tracking this sequential predictability, we compared temporal patterns between patients and healthy controls to characterize overall discourse trajectories and pinpoint specific phases where significant linguistic deviations emerge. Furthermore, we evaluated these trajectories across diverse elicitation tasks to examine how varying cognitive and contextual demands influence dynamic speech patterns. We hypothesized that while the initial context-building phase immediately following speech onset would demonstrate comparable surprisal between groups, patients would exhibit a progressively divergent, elevated surprisal trajectory as the utterance lengthens, indicative of a dynamic breakdown in contextual coherence.

## Materials and methods

### Participants

The current study analyzed speech samples obtained from a larger clinical cohort established to investigate the multidimensional linguistic features of schizophrenia. From this patient cohort, we included 249 individuals diagnosed with schizophrenia spectrum disorders and 159 healthy controls, group-matched for age and sex. Patients were recruited from community mental health centers and psychiatric outpatient clinics. Patients were included if they were between 19 and 59 years of age. Additionally, they were required to have a primary diagnosis of Schizophrenia Spectrum and Other Psychotic Disorders, according to the Diagnostic and Statistical Manual of Mental Disorders, Fifth Edition (DSM-5). The control group comprised individuals within the same age range, with no history of any psychiatric disorders. Participants with severe physical illnesses or those unable to provide valid informed consent were excluded from both groups.

Clinical status, symptom severity, and psychosocial functioning were evaluated using the Positive and Negative Syndrome Scale (17), Clinical Global Impression-Schizophrenia (18), and Social and Occupational Functioning Assessment Scale (19). The Institutional Review Board of Chonnam National University Hospital approved the study protocol (CNUH-2024-315), and all participants provided written informed consent.

### Speech data acquisition and preprocessing

Speech samples were collected using a standardized protocol comprising eight distinct tasks designed to elicit natural speech across diverse contexts (**Supplementary Table 1**). The elicitation protocol consisted of three domains. First, the Free Narrative domain assessed daily life narratives and recall, asking participants to recount a memorable event from the past week (Week), describe their daily routine (Day), and summarize a recent movie or video plot (TV). Second, the Emotional Narrative domain elicited speech with specific emotional valence, requiring descriptions of memories associated with happiness/gratitude (Happy) or anger/distress (Angry). Third, the Projective Narrative domain used three color photographs to assess descriptive and projective skills. Participants were shown images of a puppy in a cup (Picture 1; positive), gravel stones (Picture 2; neutral), and a fire scene (Picture 3; negative), and were instructed to describe their thoughts and construct a related story.

All sessions were audio-recorded, with participants instructed to sustain speech for a target duration of one minute per task. If a participant stopped speaking prematurely, the examiner provided a standardized prompt (e.g., “Could you tell me more?”) to encourage continuation; recordings were paused during these prompts to minimize interference. Subsequently, audio files were transcribed using Vrew (VoyagerX Inc., Seoul, South Korea), an AI-based automated speech recognition tool. All transcripts were manually reviewed and normalized according to a standardized protocol to ensure linguistic consistency while preserving clinically relevant speech features (**Supplementary Table 2**).

For computational modeling, speech samples were required to meet a minimum length criterion of 50 tokens to ensure sufficient context for trajectory analysis.

### Language models and token-level surprisal calculation

To calculate sequential token probabilities, we employed two open-source Korean Large Language Models (LLMs): Polyglot-ko-5.8b (Hugging Face ID: EleutherAI/polyglot-ko-5.8b; released late 2022) (20) and Kanana-1.5-8b-base (Hugging Face ID: kakaocorp/kanana-1.5-8b-base; released early 2025) (21). We deliberately selected base models rather than instruction-tuned models to capture the fundamental statistical properties of the language without the bias of task-specific fine-tuning. Furthermore, these 7B-class models were chosen to optimally balance linguistic performance with available computational resources. Both models utilize a GPT-style, decoder-only transformer architecture pre-trained on extensive Korean text corpora, enabling the autoregressive calculation of next-token probabilities based strictly on unidirectional prior context. To maximize computational precision during inference, both models were loaded in 16-bit floating-point format (FP16) without quantization.

We extracted token-level surprisal to map the dynamic predictability of speech over time. Surprisal serves as a probabilistic metric of the model’s uncertainty at each specific step, defined as the negative log-likelihood of a target token conditioned solely on its preceding context (11, 13). Formally, for a sequence of tokens $X\;=\;\left(x_{1},\;\ldots ,\;x_{N}\right)$, the surprisal at position *i* is calculated as:


$$\text{Surprisal}\left(x_{i}\right)=-\ln P_{\theta }\left(x_{i}\;|\;x_{<i}\right)$$

where *P_θ_* denotes the conditional probability assigned by the model parameters *θ* to the *i*-th token, given the preceding context $x_{<i}=\;\left(x_{1},\;x_{2},\;\ldots ,\;x_{i-1}\right)$ (22). This metric was computed sequentially for each token from position 1 to 100, constructing a discrete temporal trajectory of linguistic predictability for every speech sample. However, to control for the inherent probabilistic instability of autoregressive models at the immediate onset of speech, where predictions are highly volatile due to a lack of accumulated prior context (23), the first 10 tokens (positions 1–10) were excluded from the subsequent statistical trajectory analyses.

### Statistical analysis

Demographic and clinical characteristics were compared using the Mann-Whitney *U* test for continuous variables and the Chi-square test for categorical variables. To appropriately analyze the non-linear temporal trajectories of surprisal across token positions, we utilized Generalized Additive Mixed Models (GAMMs) fitted with fast restricted maximum likelihood (fREML) estimation (24). As natural speech lengths varied across participants, sequences shorter than the 100-token maximum were treated as unbalanced longitudinal data with missing values at later positions. GAMMs are highly robust for such sequential and temporally correlated datasets, allowing for the flexible modeling of complex non-linear curves while inherently accommodating the unbalanced longitudinal data resulting from varying utterance lengths (25).

In these models, token-level surprisal spanning positions 11 through 100 was entered as the dependent variable. To isolate the independent effect of the diagnosis, the models included diagnostic group as the primary predictor, while adjusting for sex, age, years of education, and total token count (to control for individual differences in overall speech verbosity) as parametric covariates. Non-linear smooth terms (basis dimension k = 15) were estimated for token position interacting with the diagnostic group, permitting the shape of the surprisal trajectory to vary dynamically between patients and controls over the course of the utterance. Furthermore, factor-smooth interactions for participant ID were incorporated as random effects to account for inter-individual variability in baseline predictability and individualized trajectory shapes. Following model fitting, difference smooths were computed to statistically compare the estimated trajectories of the two groups. This technique calculates the point-wise difference between the patient and control smooth curves and generates conservative 95% confidence intervals by incorporating smoothing parameter uncertainty, enabling us to pinpoint the exact token intervals where the surprisal trajectories significantly diverged (25). Statistical significance was defined as a two-tailed *P* < 0.05.

All computational procedures for data preprocessing and token-level surprisal extraction were conducted using Python (v3.10.19). Language models were implemented utilizing PyTorch (26) and the Hugging Face Transformers library (27), while data structuring was handled via Pandas (28) and NumPy (29). Subsequent statistical evaluations, specifically the GAMM analyses, were performed in the R statistical computing environment (v4.4.3) utilizing the mgcv (24) and itsadug (30) packages for model estimation and the visualization of difference smooths.

## Results

### Demographic and clinical characteristics

**Table 1** details the demographic and clinical profiles of the entire study population (n = 249 patients; n = 159 healthy controls). The two cohorts were well-matched regarding age (*U* = 20889.50, *P* = 0.346) and sex distribution (*χ²* < 0.01, *P* = 1.000). However, educational attainment was significantly lower in the patient group (*U* = 10569.50, *P* < 0.001). The patient sample was generally characterized by a chronic illness trajectory with moderate symptom severity.

**Table 1:** Demographic and clinical characteristics of the total study sample.

Variables	Patient (n = 249)	Control (n = 159)	Statistics
Age, years	35.00 (29.00, 44.00)	34.00 (30.00, 41.00)	*U* = 20889.50, *P* = 0.346
Sex (male / female), n	117 / 132	75 / 84	*χ^2^*^<^ 0.01, *P* = 1.000
Years of education	14.00 (12.00, 16.00)	16.00 (15.00, 16.00)	*U* = 10569.50, *P* < 0.001
Marital status, n^a^	26 / 0 / 209 / 12 / 2	80 / 4 / 72 / 3 / 0	*χ^2^* = 90.24, *P* < 0.001
Employment status, n^b^	28 / 14 / 16 / 31 / 160	98 / 10 / 18 / 6 / 27	*χ^2^* = 134.02, *P* < 0.001
Diagnosis, n^c^	221 / 5 / 3 / 20	–	*-*
Duration of illness, years	10.00 (5.00, 17.00)	–	–
Antipsychotic dose, mg/day^d^	200.00 (133.00, 432.55)	–	–
PANSS Total	50.00 (42.00, 56.50)	–	–
CGI-SCH Overall severity	3.00 (3.00, 4.00)	–	–
SOFAS	55.00 (55.00, 65.00)		

Data are presented as median (interquartile range) or number. Statistical comparisons were performed using the Mann-Whitney U test for continuous variables and the Chi-square test for categorical variables, respectively.

^a^ Data are presented as married / cohabiting / single (never married) / divorced / widowed.

^b^ Data are presented as full-time / self-employed / temporary (full-time) / part-time / unemployed.

^c^ Data are presented as number of patients with schizophrenia / schizoaffective disorder / schizophreniform disorder / other psychotic disorders.

^d^ Antipsychotic dosage was converted into chlorpromazine equivalents.

PANSS, Positive and Negative Syndrome Scale; CGI-SCH, Clinical Global Impression-Schizophrenia; SOFAS, Social and Occupational Functioning Assessment Scale.

**Figure 1:**
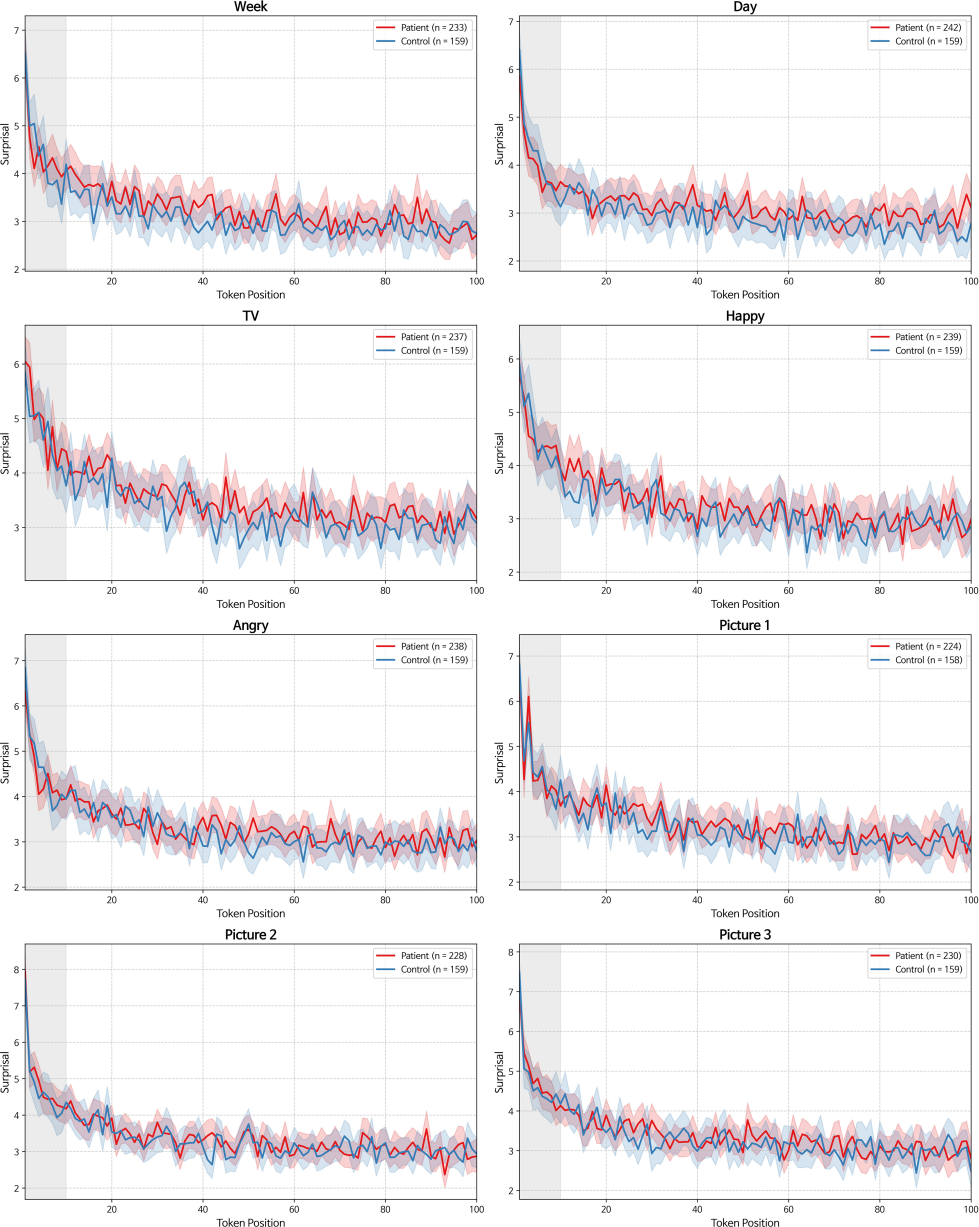
Sequential trajectories of token-level surprisal across eight speech tasks using the Polyglot-ko-5.8b model. The line plots illustrate the dynamic predictability of speech over 100 token positions. Red lines represent patients with schizophrenia, and blue lines represent healthy controls. The colored shaded regions around the lines denote the 95% confidence intervals. The vertical gray shaded areas (tokens 1–10) indicate the initial phase of maximum probabilistic uncertainty, which was excluded from the statistical trajectory analyses.

**Figure 2:**
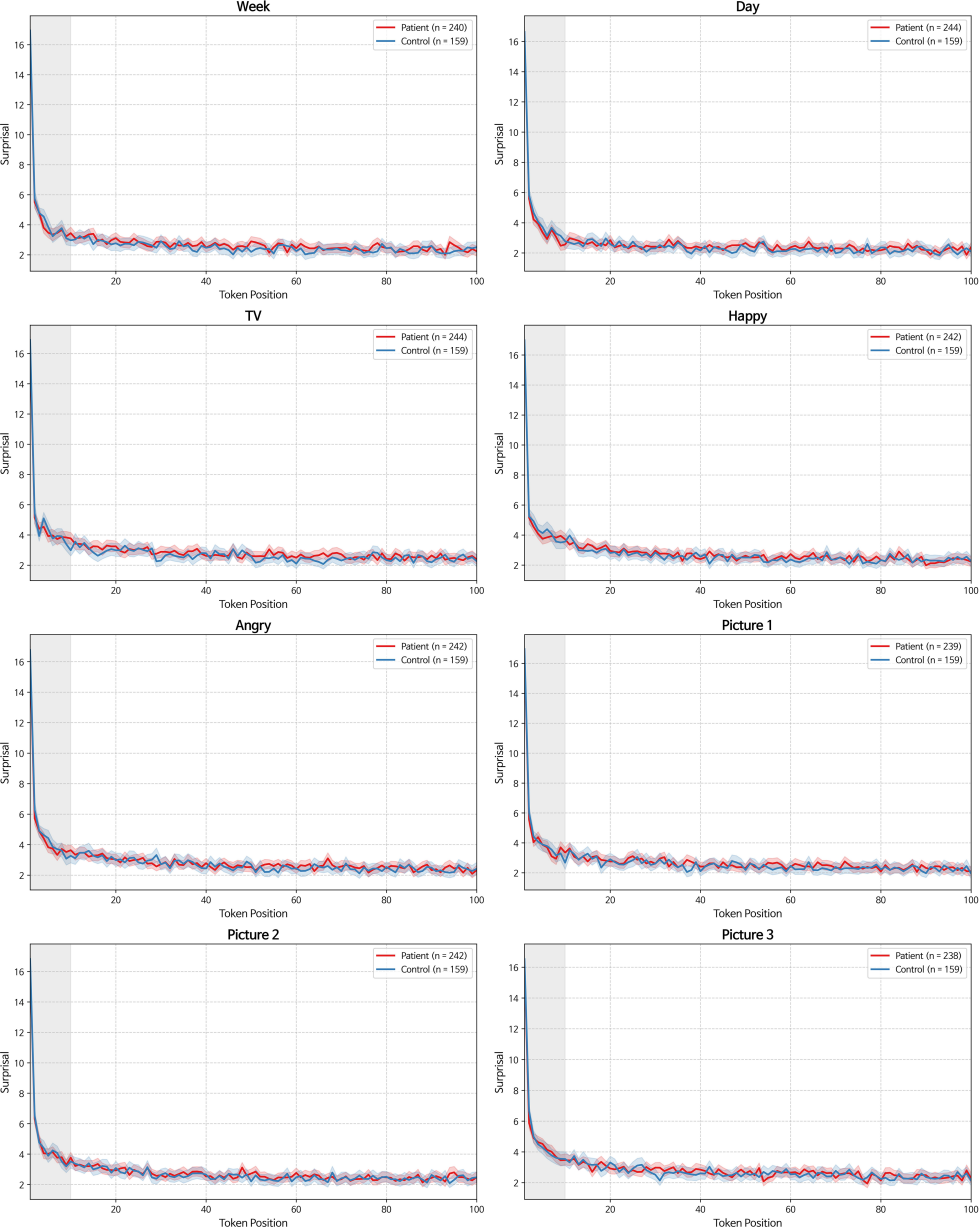
Sequential trajectories of token-level surprisal across eight speech tasks using the Kanana-1.5-8b-base model. The line plots illustrate the dynamic predictability of speech over 100 token positions. Red lines represent patients with schizophrenia, and blue lines represent healthy controls. The colored shaded regions around the lines denote the 95% confidence intervals. The vertical gray shaded areas (tokens 1–10) indicate the initial phase of maximum probabilistic uncertainty, which was excluded from the statistical trajectory analyses.

For the computational analyses, valid subsamples were established using a minimum threshold of 50 tokens per task. Due to variations in tokenizer algorithms, the final number of included samples differed slightly between the two language models (Polyglot-ko-5.8b and Kanana-1.5-8b-base); specific sample sizes per task and model are detailed in **Figures 1**, **2**. Importantly, the demographic composition within these task-specific subsets aligned closely with the total sample, confirming that filtering by response length did not induce selection bias (**Supplementary Tables 3**, **4**).

### General trajectories of token-level surprisal

**Figures 1**, **2** illustrate the sequential trajectories of token-level surprisal generated by the Polyglot-ko-5.8b and Kanana-1.5-8b-base models, respectively, across the eight speech tasks. Both models revealed a highly consistent structural pattern of discourse predictability. Specifically, surprisal was highest at the immediate onset of the utterances (approximately tokens 1 to 10). This initial peak reflects the maximum probabilistic uncertainty inherent to unidirectional language models at the beginning of an utterance, where next-token predictions must be made before the accumulation of a robust prior context. Following this peak, surprisal values demonstrated a gradual, logarithmic-like decline, eventually stabilizing as the accumulating context facilitated more predictable next-token generation.

### Temporal divergence of surprisal

**Figure 3:**
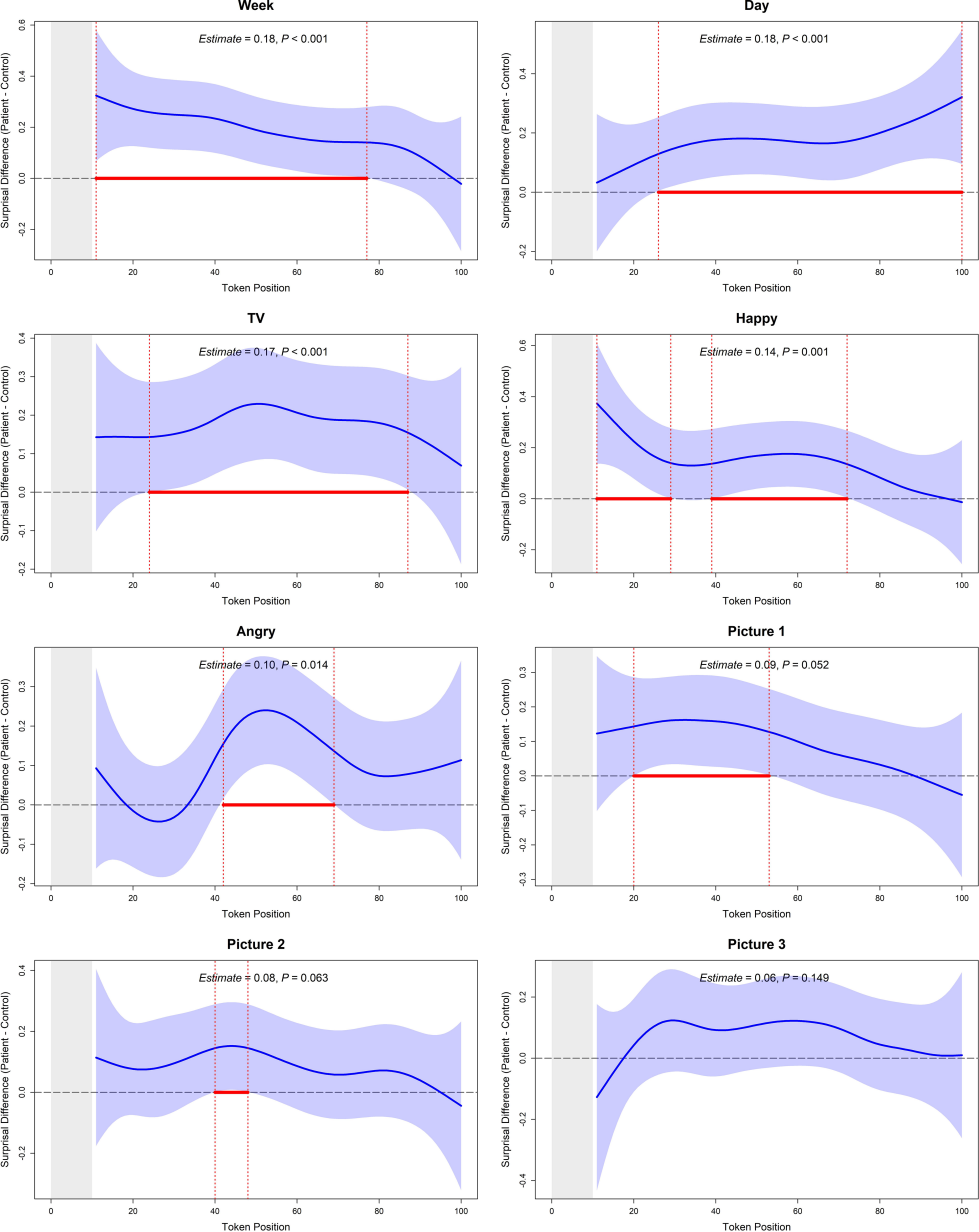
Generalized additive mixed model (GAMM)-derived difference smooths of surprisal trajectories using the polyglot-ko-5.8b model. The solid blue line represents the point-wise estimated difference in surprisal between the groups (Patient – Control) across token positions, with the shaded blue regions indicating 95% confidence intervals. The horizontal black line at zero represents no statistical difference between the groups. The bold red horizontal lines highlight the specific temporal intervals where the surprisal trajectories of the two groups significantly diverge (i.e., the 95% confidence interval does not overlap with zero). The vertical gray shaded areas (tokens 1–10) indicate the initial phase of maximum probabilistic uncertainty, which was excluded from the statistical trajectory analyses. The overall estimate and *P*-value for the mean surprisal difference are provided for each task.

**Figure 4:**
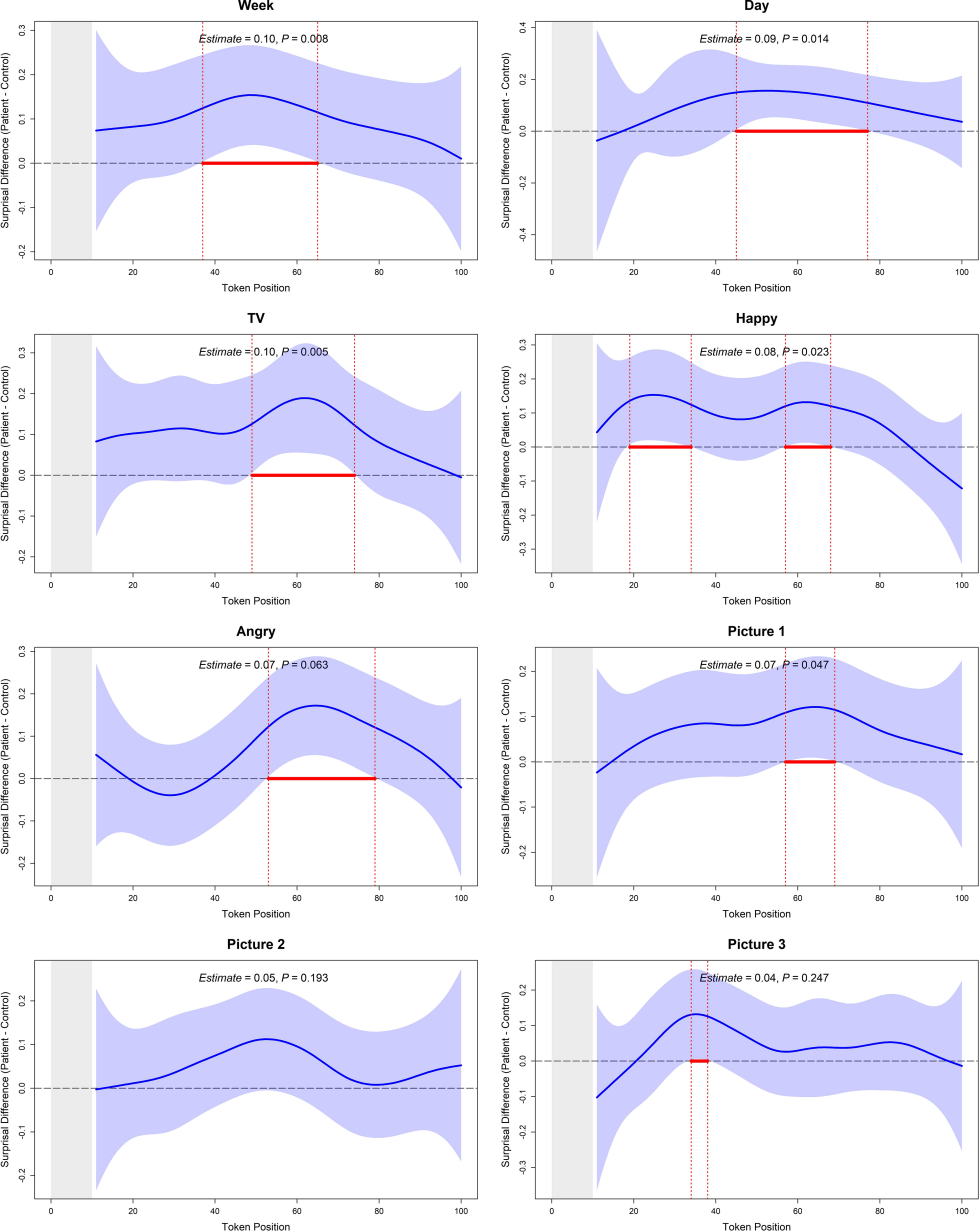
Generalized additive mixed model (GAMM)-derived difference smooths of surprisal trajectories using the Kanana-1.5-8b-base model. The solid blue line represents the point-wise estimated difference in surprisal between the groups (Patient – Control) across token positions, with the shaded blue regions indicating 95% confidence intervals. The horizontal black line at zero represents no statistical difference between the groups. The bold red horizontal lines highlight the specific temporal intervals where the surprisal trajectories of the two groups significantly diverge (i.e., the 95% confidence interval does not overlap with zero). The vertical gray shaded areas (tokens 1–10) indicate the initial phase of maximum probabilistic uncertainty, which was excluded from the statistical trajectory analyses. The overall estimate and *P*-value for the mean surprisal difference are provided for each task.

To pinpoint the specific temporal intervals where linguistic predictability significantly deviated between groups, we analyzed GAMM-derived difference smooths (**Figures 3**, **4**). This primary analysis revealed that the contextual disruption in schizophrenia is not a uniform, static feature but emerges dynamically at distinct phases of discourse.

In the Polyglot-ko-5.8b model, significant divergence intervals were observed across seven of the eight tasks, except for Picture 3 (**Figure 3**). In free narrative tasks, linguistic deviations were remarkably broad and intensified after the initial context-building phase. The Week task showed a continuous, extensive divergence from tokens 11 to 77. Similarly, the Day task exhibited a prolonged interval of significant deviation spanning the remainder of the discourse (tokens 26–100), while the TV task diverged significantly from tokens 24 to 87. Emotional narratives (Happy: tokens 11–29 and 39–72; Angry: tokens 42–69) and projective picture tasks (Picture 1: tokens 20–53; Picture 2: tokens 40–48) consistently demonstrated that the most pronounced linguistic deviations occurred in the middle to late phases of the utterances.

The Kanana-1.5-8b-base model yielded fewer significant intervals than the Polyglot model but confirmed a similar trajectory, characterized by robust, consolidated deviations emerging in the mid-to-late phases of the 100-token discourse (**Figure 4**). Free narrative tasks showed broad intervals of significant divergence, specifically in the Week (tokens 37–65), Day (tokens 45–77), and TV (tokens 49–74) tasks. Emotional narratives also captured distinct windows of deviation (Happy: tokens 19–34 and 57–68; Angry: tokens 53–79). In the projective picture tasks, significant temporal divergence was observed in Picture 1 (tokens 57–69) and Picture 3 (tokens 34–38). Picture 2 was the only task where no significant temporal divergence was detected by this model.

Crucially, both models revealed distinct and overlapping windows of linguistic divergence, particularly within the narrative domains. When comparing the significant intervals from both models, robust cross-task overlaps consistently emerged during the mid-to-late phases of the 100-token discourse. Specifically, these exact overlapping intervals occurred in the Week (tokens 37–65), Day (tokens 45–77), TV (tokens 49–74), and Angry (tokens 53–69) tasks. The Happy task demonstrated overlapping divergence in both an earlier window (tokens 19–29) and a later phase (tokens 57–68). In contrast, the projective picture tasks did not yield any overlapping intervals of significant deviation between the two models.

### Overall mean surprisal differences

In addition to the temporal analyses, we examined the overall mean surprisal differences between groups (**Figures 3**, **4**). In the Polyglot-ko-5.8b model, patients exhibited significantly higher overall surprisal in the Week (*P* < 0.001), Day (*P* < 0.001), TV (*P* < 0.001), Happy (*P* = 0.001), and Angry (*P* = 0.014) tasks (**Figure 3**). Marginal trends toward significance were noted for the Picture 1 (*P* = 0.052) and Picture 2 (*P* = 0.063) tasks, while the Picture 3 task did not show a significant difference (*P* = 0.149).

In the Kanana-1.5-8b-base model, the overall mean difference reached statistical significance in the Week (*P* = 0.008), Day (*P* = 0.014), TV (*P* = 0.005), Happy (*P* = 0.023), and Picture 1 (*P* = 0.047) tasks (**Figure 4**). A marginal trend was observed in the Angry task (*P* = 0.063), whereas the remaining projective picture tasks (Picture 2 and Picture 3) did not reach statistical significance (*P* = 0.193 and *P* = 0.247, respectively).

## Discussion

This study investigated the dynamic temporal trajectory of disorganized speech in a Korean-speaking cohort with schizophrenia using sequential token-level surprisal derived from autoregressive language models. Across a diverse battery of speech tasks, patients exhibited significantly divergent and elevated surprisal trajectories compared with healthy controls. Trajectory analyses using GAMMs revealed that while both groups maintained normative predictability during the initial context-building phase, patients demonstrated a progressive breakdown in contextual predictability as the discourse unfolded. Specifically, marked linguistic deviations consistently manifested and intensified during the mid-to-late phases of these short, 100-token utterances. Collectively, these findings provide robust quantitative evidence that language anomalies in schizophrenia are not uniformly distributed static features; rather, they emerge dynamically, reflecting a profound deficit in sustaining contextual continuity as the cognitive demands of discourse accumulate.

Our findings align with and extend the growing body of literature utilizing language model-derived probabilities to quantify formal thought disorder in schizophrenia (4, 31). Previous studies have demonstrated that language models, whether employing unidirectional (e.g., GPT) or bidirectional (e.g., BERT) architectures, can effectively capture the semantic and syntactic disruptions inherent in the speech of patients with schizophrenia (32–35). By measuring the predictability of lexical choices, computational metrics such as perplexity and pseudo-perplexity have successfully operationalized clinical abnormalities, correlating with the severity of formal thought disorder and effectively differentiating patients from healthy controls. Furthermore, a recent study explicitly highlighted the diagnostic utility of surprisal by demonstrating that maximum word-level surprisal values significantly enhance the automated detection of loose associations in early psychosis (36). While these prior investigations have demonstrated the potential of language models as valuable tools for clinical assessment, their reliance on static, document-level averages or aggregate summary features highlights the need to further account for the continuous, unfolding nature of natural speech. To address this, our approach shifts the analytical focus to the temporal dynamics of token-level surprisal. By evaluating predictability sequentially, we demonstrate that the linguistic divergence between patients and healthy controls is not merely a constant baseline deficit, but rather a time-dependent phenomenon. This dynamic perspective provides a crucial foundation for pinpointing the exact temporal intervals where contextual coherence breaks down.

The primary finding from our difference smooth analyses, that linguistic divergence manifests progressively as discourse unfolds, provides critical temporal resolution to the specific nature of formal thought disorder. Analyzing the trajectories up to 100 tokens, we observed that although linguistic divergence could emerge earlier depending on the model, significant and robust deviations consistently manifested and intensified between tokens 50 and 70. Given that 100 tokens comprise merely a few sentences, these cross-model overlaps indicate a rapid breakdown of contextual coherence in schizophrenia. This temporal pattern strongly corroborates recent psycholinguistic evidence regarding contextual processing in schizophrenia. Notably, Sharpe et al. demonstrated that disorganized speech is characterized by a disproportionate insensitivity to global versus local linguistic context (37). While patients’ ability to use local linguistic context remains relatively preserved, they exhibit a marked deficit in utilizing broader discourse constraints. Because the generation of the initial tokens in a sequence relies predominantly on local syntactic rules and immediate semantic associations, patients can often initiate utterances with normative predictability. However, the production of coherent language requires the continuous integration of broader contextual constraints with local dependencies (3). The robust temporal divergence we observed, emerging rapidly and consistently intensifying by tokens 50 to 70, quantitatively captures this specific breakdown just beyond the boundaries of immediate local context. It indicates that while patients successfully construct short, localized phrases, their capacity to leverage accumulated global information rapidly deteriorates. This computational pattern aligns with clinical descriptions of disorganized discourse, where patients seem to lose track of global sources of information even though local relationships within short phrases remain intact. Moreover, this divergence appears to plateau or even diminish rather than widening indefinitely beyond this point, which may be attributed to speakers repeatedly resetting their discourse with new, locally coherent phrases, thereby attenuating the significant differences in surprisal between the two groups.

In addition to the dynamic temporal analyses, our evaluation of the overall mean surprisal demonstrated significant differences between the diagnostic groups. Both the Polyglot-ko-5.8b and Kanana-1.5-8b-base models detected significantly higher global unpredictability in patients across multiple speech tasks. While our primary dynamic analyses track the progressive temporal decay of context, this global metric effectively captures the cumulative burden of pervasive contextual disruptions. Conceptually, overall surprisal captures linguistic features highly analogous to other global probabilistic indices, such as perplexity derived from unidirectional GPT-style models and pseudo-perplexity derived from bidirectional BERT-style models (11). Therefore, our findings imply that metrics like perplexity and pseudo-perplexity may also be effectively utilized to differentiate patients with schizophrenia from healthy controls. While studies using unidirectional perplexity have primarily correlated this metric with symptom severity within patient cohorts (38), successful group-level differentiation between patients and controls has mostly relied on bidirectional pseudo-perplexity (32, 35). Taken together, our current findings suggest that global predictability indices, whether measured at the sentence, utterance, or broader discourse levels, can serve as promising and objective markers for differentiating clinical populations, extending the diagnostic utility of these computational approaches.

The manifestation of temporal divergence in surprisal varied notably across different speech elicitation tasks. Specifically, significant linguistic deviations were consistently more prominent and robust in narrative domains (both free and emotional) compared to projective tasks. This task-dependent variation likely reflects the differing cognitive demands associated with the elicitation stimuli (39). Projective tasks provide concrete visual cues that serve as external scaffolding. This external support reduces the cognitive load required for discourse planning and helps constrain semantic deviations, allowing patients to maintain contextual coherence for longer durations (40). In contrast, free and emotional narratives lack such visual cues, demanding greater internal executive control to autonomously generate and maintain a cohesive thematic structure over time (41). Consequently, the heightened reliance on intrinsic generation mechanisms during narrative tasks accelerates the depletion of cognitive resources, precipitating a more pronounced breakdown in contextual predictability. These findings underscore the importance of utilizing a diverse task battery, as the dynamic emergence of linguistic deficits is highly sensitive to the availability of external contextual support.

Furthermore, our study employed Korean-specific smaller language models (7B-class models) rather than large-scale LLMs. This approach aligns with recent evidence suggesting that smaller models can paradoxically outperform large-scale LLMs in detecting formal thought disorder (38). We hypothesize that large-scale LLMs, often trained on vast datasets and optimized for conversational fluency, may be prone to over-smoothing, which could inadvertently normalize pathological linguistic deviations (42). In contrast, models with more constrained parameter spaces may maintain a more rigid normative baseline, retaining higher sensitivity to such deviations (38). Notably, even a 2-billion-parameter model (Gemma 2B) successfully utilized word-level surprisal to accurately detect loose associations in early psychosis (36). This possibility is consistent with our results: the older Polyglot-ko model yielded broader temporal divergence intervals, particularly capturing extensive, continuous significance across the free narrative tasks, than the newer Kanana model. This discrepancy suggests that differences in the composition of the pre-training corpora may further influence sensitivity; earlier models relying on standardized, document-based corpora might establish a rigid baseline highly sensitive to speech disorganization, whereas Kanana’s optimization for conversational fluency could inherently normalize these irregularities. While these mechanistic explanations remain speculative and warrant further systematic validation, our findings highlight that detecting dynamic linguistic deficits requires careful consideration of model architectures and their intrinsic smoothing properties.

This study has several limitations. First, truncating the temporal trajectories at 100 tokens limits the evaluation of long-range discursive deficits, warranting future studies with extended, continuous narratives. Second, despite employing robust GAMMs and controlling for total token count, intrinsic group differences in verbosity, with healthy controls typically producing longer narratives, remain a methodological challenge. Third, utilizing a customized task battery rather than standardized projective instruments (e.g., the Thematic Apperception Test) limits direct comparability with conventional protocols. Nevertheless, it facilitated language assessment across diverse cognitive and emotional loads, minimized cultural biases, and ensured task accessibility. Future studies are required to formally validate this custom battery and replicate our findings in independent cohorts. Fourth, our standardized speech protocol limits the assessment of the interactional nature of language. Minimizing examiner intervention was necessary to isolate the speaker’s internal capacity for discourse organization, but it inherently excludes the co-constructed dynamics of natural conversation. Future studies using dyadic interactions are needed to explore how these contextual disruptions manifest in real-world communication. Fifth, our findings depend on specific model architectures. Although both models identified linguistic deviations, subtle differences in their sensitivities indicate that model size and pre-training paradigms influence outcomes, necessitating further exploration across diverse architectures. Finally, although significant educational disparities between groups were statistically adjusted, residual effects on the linguistic metrics cannot be completely ruled out. Furthermore, clinical variables unique to the patient group, such as symptom severity and antipsychotic dosage, could not be controlled for in the between-group analysis. Future within-group investigations are warranted to clarify the specific influence of these clinical factors on language production.

In conclusion, this study demonstrates that linguistic anomalies in schizophrenia are dynamic phenomena rather than static deficits. By tracking sequential token-level surprisal, we revealed a progressive breakdown in contextual predictability occurring even within a few sentences. Although patients successfully initiate speech using local dependencies, their capacity to integrate global context rapidly deteriorates, a pattern that appears to parallel clinical descriptions of disorganized discourse. Pinpointing these precise intervals of contextual disruption highlights dynamic, automated language analysis as a highly sensitive and scalable tool for elucidating the neurocognitive underpinnings of formal thought disorder.

## Data Availability

The datasets presented in this article are not readily available because the datasets generated and analyzed during this study are not publicly available due to privacy and ethical restrictions regarding sensitive clinical speech data and the risk of breaching participant confidentiality. However, anonymized datasets are available from the corresponding author on reasonable request, subject to approval by the Institutional Review Board. Requests to access the datasets should be directed to Sung-Wan Kim, swkim@chonnam.ac.kr.
