# Supplementary material for: Tracking the dynamic breakdown of contextual coherence in schizophrenia using language models
Source: Front Psychiatry. 2026 Jun 1;17:1848956. doi: 10.3389/fpsyt.2026.1848956 (PMC13265483; doi:10.3389/fpsyt.2026.1848956)
Supplement: Supplementary file 1 [file Table1.docx]

| **Supplementary Table 1.** Standardized Scripts for Speech Elicitation Tasks. | | | | |
| --- | --- | --- | --- | --- |
| Domain | Task (Abbreviation) | | Standardized Instruction | |
| Free Narrative | Memorable Event (Week) | | “Please tell me about a memorable event from the past week. Since you are asked to speak for one minute, please describe it in as much detail as possible.” | |
|  | Daily Routine (Day) | | “Please describe your typical daily routine these days. Since you are asked to speak for one minute, please describe it in as much detail as possible.” | |
|  | Movie or Video Summary (TV) | | “Please tell me the plot of a movie, drama, or YouTube video you have watched recently. Since you are asked to speak for one minute, please describe it in as much detail as possible.” | |
| Emotional Narrative | Positive Emotion (Happy) | | “Please talk about an experience or memory where you felt happy or grateful. Since you are asked to speak for one minute, please describe it in as much detail as possible.” | |
|  | Negative Emotion (Angry) | | “Please talk about an experience or memory where you felt angry or distressed. Since you are asked to speak for one minute, please describe it in as much detail as possible.” | |
| Projective Narrative | Picture-Elicited Narrative (Picture 1, 2, and 3) | | “Please describe any thoughts or feelings that come to mind while looking at this picture. Then, please try to construct a story related to the picture. Since you are asked to speak for one minute, please describe it in as much detail as possible.” | |
| 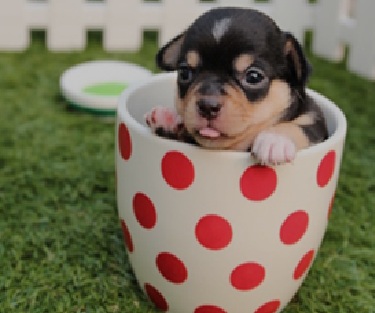Picture 1 | | 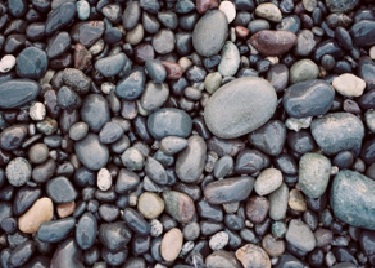Picture 2 | | 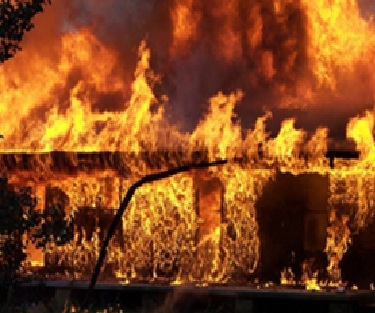Picture 3 |
| Note: The original instructions were administered in Korean to native Korean speakers. The English translations provided above are for illustrative purposes to describe the study protocol. For all tasks, if the participant ceased speech before the one-minute target duration, the examiner provided a standardized prompt (e.g., “Could you tell me more?”) to encourage continuation while minimizing interference. The recording was paused during the examiner's intervention. | | | | |

| **Supplementary Table 2.** Text Processing and Normalization Protocol. | |
| --- | --- |
| Non-lexical Artifacts | Acoustic artifacts (e.g., laughter, coughing) and all interviewer prompts were removed to isolate patient speech |
| Linguistic Features | Clinically relevant features such as fillers and hesitations were retained to preserve the natural characteristics of disorganized speech |
| Orthographic Normalization | Numbers and foreign loanwords were orthographically normalized into standard Korean script, and overt errors in spelling or spacing were manually corrected to minimize probabilistic bias. |

| **Supplementary Table 3.** Demographic characteristics and token counts for task-specific subsamples^a^ included in the analysis using Polyglot-ko-5.8b. | | | | | | | | |
| --- | --- | --- | --- | --- | --- | --- | --- | --- |
| Groups | Week  (233 / 159)^b^ | Day  (242 / 159) | TV  (237 / 159) | Happy  (239 / 159) | Angry  (238 / 159) | Picture 1  (224 / 158) | Picture 2  (228 / 159) | Picture 3  (230 / 159) |
| Age, years | | | | | | | | |
| Patient | 34.00 (29.00, 43.00) | 35.00 (29.00, 44.00) | 34.00 (29.00, 44.00) | 34.00 (29.00, 43.00) | 34.00 (29.00, 43.00) | 34.00 (29.00, 43.00) | 34.00 (29.00, 43.00) | 34.00 (29.00, 43.00) |
| Control | 34.00 (30.00, 41.00) | 34.00 (30.00, 41.00) | 34.00 (30.00, 41.00) | 34.00 (30.00, 41.00) | 34.00 (30.00, 41.00) | 33.50 (30.00, 40.75) | 34.00 (30.00, 41.00) | 34.00 (30.00, 41.00) |
| Statistic | *U* = 18926.00, *P* = 0.715 | *U* = 20156.00, *P* = 0.419 | *U* = 19494.50, *P* = 0.559 | *U* = 19563.00, *P* = 0.617 | *U* = 19468.50, *P* = 0.625 | *U* = 17893.00, *P* = 0.853 | *U* = 18174.00, *P* = 0.965 | *U* = 18683.50, *P* = 0.715 |
| Sex (male / female), n | | | | | | | | |
| Patient | 109 / 124 | 113 / 129 | 114 / 123 | 114 / 125 | 113 / 125 | 103 / 121 | 104 / 124 | 106 / 124 |
| Control | 75 / 84 | 75 / 84 | 75 / 84 | 75 / 84 | 75 / 84 | 75 / 83 | 75 / 84 | 75 / 84 |
| Statistic | *χ^2^* < 0.01, *P* = 1.000 | *χ^2^* < 0.01, *P* = 1.000 | *χ^2^* = 0.01, *P* = 0.937 | *χ^2^* < 0.01, *P* = 0.999 | *χ^2^* < 0.01, *P* = 1.000 | *χ^2^* = 0.03, *P* = 0.855 | *χ^2^* = 0.04, *P* = 0.843 | *χ^2^* = 0.01, *P* = 0.915 |
| Years of education | | | | | | | | |
| Patient | 14.00 (12.00, 16.00) | 14.00 (12.00, 16.00) | 14.00 (12.00, 16.00) | 14.00 (12.00, 16.00) | 14.00 (12.00, 16.00) | 14.00 (12.00, 16.00) | 14.00 (12.00, 16.00) | 14.00 (12.00, 16.00) |
| Control | 16.00 (15.00, 16.00) | 16.00 (15.00, 16.00) | 16.00 (15.00, 16.00) | 16.00 (15.00, 16.00) | 16.00 (15.00, 16.00) | 16.00 (15.00, 16.00) | 16.00 (15.00, 16.00) | 16.00 (15.00, 16.00) |
| Statistic | *U* = 10253.00, *P* < 0.001 | *U* = 10486.00, *P* < 0.001 | *U* =10200.00, *P* < 0.001 | *U* = 10415.00, *P* < 0.001 | *U* = 10298.00, *P* < 0.001 | *U* = 9722.50, *P* < 0.001 | *U* = 10047.50, *P* < 0.001 | *U* = 10192.50, *P* < 0.001 |
| Token count, n | | | | | | | | |
| Patient | 114.00 (84.00, 149.00) | 124.00 (95.25, 170.75) | 121.00 (92.00, 172.00) | 123.00 (91.00, 167.00) | 139.00 (99.25, 184.75) | 112.50 (82.75, 145.50) | 111.50 (86.00, 145.75) | 117.00 (90.00, 154.75) |
| Control | 153.00 (128.00, 177.50) | 163.00 (135.00, 185.00) | 157.00 (134.00, 178.00) | 160.00 (132.50, 185.50) | 169.00 (139.50, 193.00) | 146.50 (120.25, 168.00) | 145.00 (123.00, 169.00) | 149.00 (119.00, 178.00) |
| Statistic | *U* = 10264.50, *P* < 0.001 | *U* = 12863.50, *P* < 0.001 | *U* = 12919.00, *P* < 0.001 | *U* = 12382.50, *P* < 0.001 | *U* = 13575.00, *P* < 0.001 | *U* = 11145.50, *P* < 0.001 | *U* = 11201.00, *P* < 0.001 | *U* = 12484.50, *P* < 0.001 |
| Data are presented as median (interquartile range) or number. Group differences in demographic variables were assessed using the Chi-square test (sex) or Mann-Whitney U test (age, years of education, and token count).  ^a^ Subsamples vary by task due to the minimum 50-token threshold requirement for analysis.  ^b^ Numbers in parentheses represent the number of participants (n) in the patient and healthy control groups, respectively. | | | | | | | | |

| **Supplementary Table 4.** Demographic characteristics and token counts for task-specific subsamples^a^ included in the analysis using Kanana-1.5-8b-base. | | | | | | | | |
| --- | --- | --- | --- | --- | --- | --- | --- | --- |
| Groups | Week  (240 / 159)^b^ | Day  (244 / 159) | TV  (244 / 159) | Happy  (242 / 159) | Angry  (242 / 159) | Picture 1  (239 / 159) | Picture 2  (242 / 159) | Picture 3  (238 / 159) |
| Age, years | | | | | | | | |
| Patient | 34.50 (29.00, 43.00) | 35.00 (29.00, 44.00) | 34.50 (29.00, 44.00) | 34.00 (29.00, 43.00) | 34.00 (29.00, 43.75) | 34.00 (29.00, 43.00) | 34.00 (29.00, 43.00) | 34.00 (29.00, 43.00) |
| Control | 34.00 (30.00, 41.00) | 34.00 (30.00, 41.00) | 34.00 (30.00, 41.00) | 34.00 (30.00, 41.00) | 34.00 (30.00, 41.00) | 34.00 (30.00, 41.00) | 34.00 (30.00, 41.00) | 34.00 (30.00, 41.00) |
| Statistic | *U* = 19800.50, *P* = 0.523 | *U* = 20345.00, *P* = 0.407 | *U* = 20269.00, *P* = 0.446 | *U* = 19892.50, *P* = 0.565 | *U* = 19995.50, *P* = 0.505 | *U* = 19588.50, *P* = 0.601 | *U* = 19900.50, *P* = 0.560 | *U* = 19474.00, *P* = 0.622 |
| Sex (male / female), n | | | | | | | | |
| Patient | 112 / 128 | 114 / 130 | 116 / 128 | 114 / 128 | 114 / 128 | 111 / 128 | 115 / 127 | 110 / 128 |
| Control | 75 / 84 | 75 / 84 | 75 / 84 | 75 / 84 | 75 / 84 | 75 / 84 | 75 / 84 | 75 / 84 |
| Statistic | *χ^2^* < 0.01, *P* = 1.000 | *χ^2^* < 0.01, *P* = 1.000 | *χ^2^* < 0.01, *P* = 1.000 | *χ^2^* < 0.01, *P* = 1.000 | *χ^2^* < 0.01, *P* = 1.000 | *χ^2^* < 0.01, *P* = 0.968 | *χ^2^* < 0.01, *P* = 1.000 | *χ^2^* = 0.01, *P* = 0.933 |
| Years of education | | | | | | | | |
| Patient | 14.00 (12.00, 16.00) | 14.00 (12.00, 16.00) | 14.00 (12.00, 16.00) | 14.00 (12.00, 16.00) | 14.00 (12.00, 16.00) | 14.00 (12.00, 16.00) | 14.00 (12.00, 16.00) | 14.00 (12.00, 16.00) |
| Control | 16.00 (15.00, 16.00) | 16.00 (15.00, 16.00) | 16.00 (15.00, 16.00) | 16.00 (15.00, 16.00) | 16.00 (15.00, 16.00) | 16.00 (15.00, 16.00) | 16.00 (15.00, 16.00) | 16.00 (15.00, 16.00) |
| Statistic | *U* = 10408.50, *P* < 0.001 | *U* = 10504.00, *P* < 0.001 | *U* = 10524.50, *P* < 0.001 | *U* = 10434.00, *P* < 0.001 | *U* = 10406.00, *P* < 0.001 | *U* = 10286.00, *P* < 0.001 | *U* = 10434.00, *P* < 0.001 | *U* = 10457.50, *P* < 0.001 |
| Token count, n | | | | | | | | |
| Patient | 137.00 (101.00, 180.25) | 154.50 (121.00, 209.00) | 150.00 (111.75, 206.00) | 146.00 (111.25, 206.50) | 163.00 (118.25, 214.50) | 137.00 (99.50, 183.50) | 134.50 (99.00, 180.00) | 141.50 (104.00, 187.00) |
| Control | 183.00 (156.50, 213.00) | 198.00 (161.50, 228.00) | 190.00 (158.50, 214.50) | 187.00 (154.00, 221.50) | 197.00 (166.00, 232.00) | 181.00 (149.00, 211.50) | 178.00 (148.50, 205.50) | 176.00 (141.00, 212.50) |
| Statistic | *U* = 10656.50, *P* < 0.001 | *U* = 13472.50, *P* < 0.001 | *U* = 13578.50, *P* < 0.001 | *U* = 13027.00, *P* < 0.001 | *U* = 13848.50, *P* < 0.001 | *U* = 11644.50, *P* < 0.001 | *U* = 11592.00, *P* < 0.001 | *U* = 12770.00, *P* < 0.001 |
| Data are presented as median (interquartile range) or number. Group differences in demographic variables were assessed using the Chi-square test (sex) or Mann-Whitney U test (age, years of education, and token count).  ^a^ Subsamples vary by task due to the minimum 50-token threshold requirement for analysis.  ^b^ Numbers in parentheses represent the number of participants (n) in the patient and healthy control groups, respectively. | | | | | | | | |
